# Stock-based detection of protein oligomeric states in jsPISA

**DOI:** 10.1093/nar/gkv314

**Published:** 2015-04-23

**Authors:** Eugene Krissinel

**Affiliations:** CCP4, Research Complex at Harwell, Didcot, OX11 0FA, UK

## Abstract

A new version of the popular software *PISA* for the analysis of macromolecular interfaces and identification of biological assemblies (complexes) from macromolecular crystal structures is presented. The new web server *jsPISA* has a substantially improved user interface, based on modern JavaScript technologies, and also new elements of analysis: assembly stock and interaction radar. The new elements help interpretation of *PISA* results in difficult and ambiguous cases, for example, when the oligomeric state depends on protein concentration, or when the biologically relevant interaction is weak and cannot be easily discriminated from superficial crystal contacts. *jsPISA* is maintained by CCP4 at http://www.ccp4.ac.uk/pisa. There are no login requirements for using the server.

## INTRODUCTION

The identification of biological interactions and complexes follows crystallographic structure solution and, in many cases, is one of the main goals of crystallographic studies. This problem is complicated because, often, macromolecular complexes possess crystallographic symmetry and, therefore, cannot be easily identified in crystal lattice. On the other hand, the asymmetric unit (AU) of a crystal is normally chosen from crystallographic, rather than biological, considerations and does not always present biological units or biologically significant interactions. In addition, the biological assembly may be either larger or smaller than the AU. The *PISA* (Protein Interactions, Surfaces and Assemblies) software (1) is one of a number of tools (2–5) aimed at providing a quantified approach to solving this problem and, ultimately, to automate it.

*PISA* was deployed as a web server at the European Bioinformatics Institute (Cambridge, UK) in 2007 and since then it became a popular tool for the analysis of macromolecular structures and interactions. Shortly after its release, *PISA* became a part of the structure deposition pipeline at the Protein Data Bank (PDB) (6), where it is now routinely used for validation and annotation of *REMARK 350* records.

In most cases (the 7-year history of usage and feedback suggests a figure close to 90%), *PISA* results agree with experimental evidence on oligomeric states. The same figure holds for protein quaternary structures (PQS) where they are confirmed by complementary techniques. Errors in *PISA* are due to a variety of factors, ranging from approximations employed to differences between chemical environments in the living cell and the experimental conditions (due to the addition of crystallization agents and high protein concentration in crystallization buffer). A detailed analysis of *PISA* errors suggests that the likelihood of errors decreases exponentially with increase in the strength of macromolecular interactions within the assembly (7). In general, weakly bound complexes are more prone to dissociation and, therefore, a considerable fraction of errors should be linked to the variability of oligomeric states with protein concentration. Indeed, a typical *PISA* error is seen as a discrepancy between the PQS inferred from the crystalline state (highest possible concentration) and the oligomeric state identified by gel-filtration (measured at lower concentration).

In this paper, a new web server for the analysis of macromolecular interactions and identification of PQS from crystalline states, *jsPISA*, is presented, in which protein concentration is added as an additional parameter in the analysis. This modification changes the way a researcher should interpret results, as described below. *jsPISA* also introduces an interaction radar for scoring macromolecular interfaces, which is based on the statistical analysis of all interfaces found in the PDB. Another motivation for the development of *jsPISA* was the complete refurbishment of the graphical front end and making it more interactive and user-friendly. New features, introduced in *jsPISA*, are discussed in the following sections.

## NEW FEATURES

### Assembly stock

In *PISA*, the probable oligomeric state is identified solely on the basis of the Gibbs free energy of dissociation (Δ*G*_0_) of candidate assemblies. The candidate assemblies are obtained as sub-graphs of the crystal lattice, where the sub-graph search is performed with respect to the similarity of both graph nodes (i.e. macromolecules) and graph edges (macromolecular interfaces or interactions) (1). In most cases, the higher the Δ*G*_0_, the higher the concentration of the respective assembly in solution. However, this may not hold in complex situations, where chemical equilibrium is a result of several competing processes.

As an alternative approach, *jsPISA* introduces the *assembly stock*, defined as a thermally equilibrated solution of macromolecular assemblies, obtained by the dissolution of a crystal, and defines the most probable oligomeric state as one with the maximum aggregation index in the stock:

(1) \begin{equation*} X_i = \frac{{c_i m_i }}{{\sum\nolimits_j {c_j m_j } }} \end{equation*}

In Equation (1), *c_i_* is concentration, and *m_i_* – oligomeric state expressed as the number of monomeric units (macromolecular chains) of the *i*th assembly. The aggregation index varies between 0 (oligomeric state not present in solution) and 1 (fully aggregated in one state). The advantage of this *X*-score is in that it indicates the fraction of monomeric units aggregated in a particular oligomeric state, rather than a mere comparison of concentrations. This is more convenient than, e.g. mole fraction, in situations when a large assembly (such as a viral capsid with *m_a_* ≈ 300–700 or higher) is equilibrated with free monomeric chains (*m_f_* = 1); for as long as $c_f /c_a < m_a /m_j$, the aggregation index will show that most of the protein mass is in the aggregated state, while the mole fraction would indicate that the concentration of free chains is higher than that of capsids.

### Interaction radar

**Figure 1.**
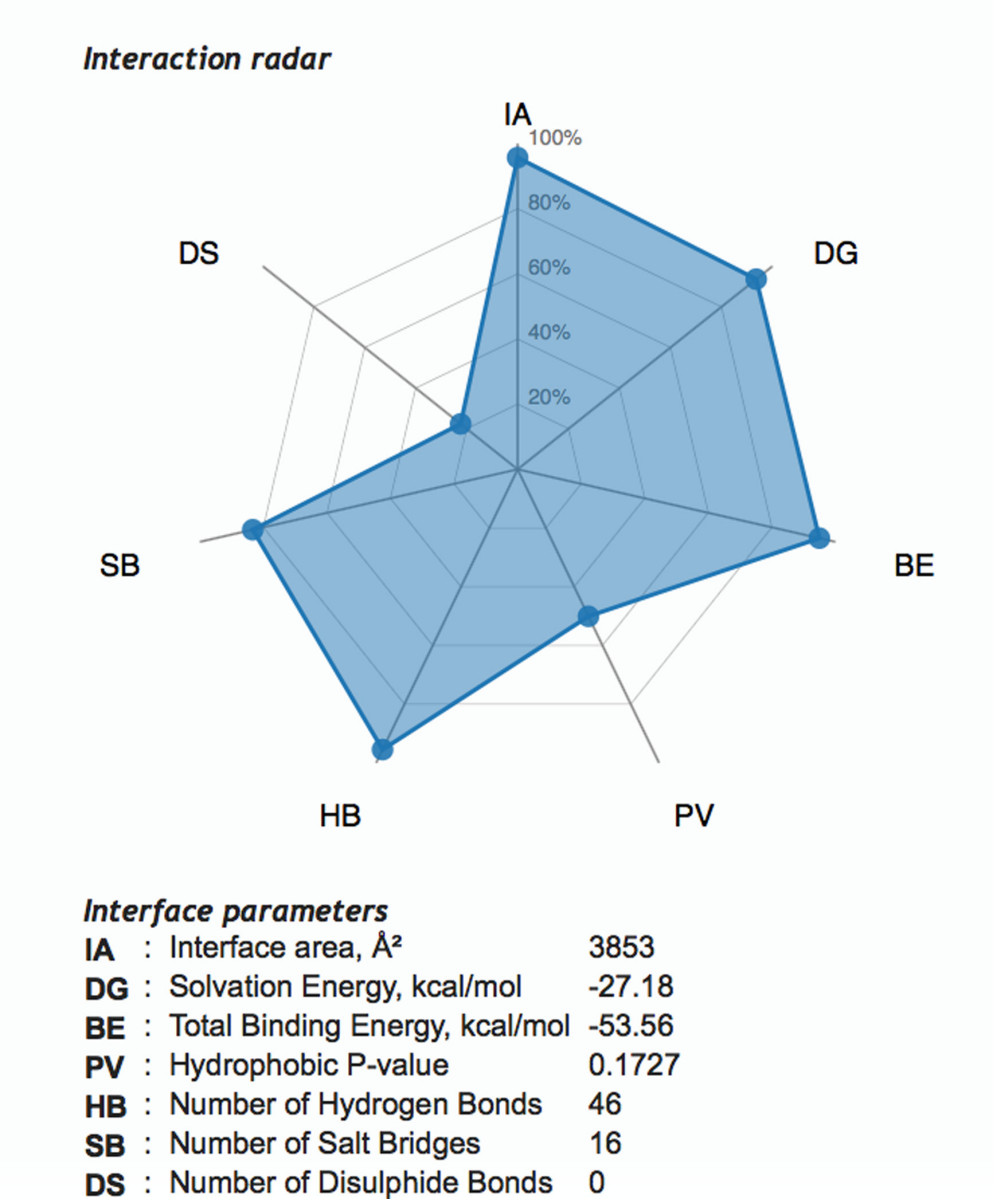
Screen shot of interaction radar in jsPISA.

It is often assumed that biologically significant interactions manifest themselves as crystal contacts. Usually, discrimination between significant contacts and artifacts of crystal packing is difficult, and various bioinformatics techniques are employed to aid this (see, e.g. (2) and discussion in (7)). It has been established that such discrimination cannot be achieved with a single score (2) despite numerous attempts performed to date (see (1,7) and references therein). Potentially, a combined score could be produced utilizing a hash function that maps a few (5–7) major interface parameters to the likelihood of that interface being a part of the biological assembly. However, the current size of the PDB is not sufficient by far to gather reliable statistics in the multi-parameter space (e.g. if five parameters are chosen, then the ∼10^5^ PDB entries of today can provide only for a very sparse grid of 10 counts per parameter, which is not sufficient for further boxing and likelihood calculations. In reality, the grid is effectively much sparser owing to the high level of redundancy in the PDB). Therefore, *jsPISA* offers an alternative route of interface scoring using the interaction radar (Figure 1). The radar consists of seven beams, representing seven interface parameters directly related to interface binding properties (cf. Figure 1). The scale of each beam indicates the probability of the interface with the corresponding parameter being a part of the biological assembly, with zero probability placed at radar's origin. This design makes clear the correlation between radar area and the biological significance of the interface: the larger the area, the higher the likelihood of finding the interface within the biological assemblage. If all of the radar area fits within the 50% probability circle, then the interface is likely to be a superficial crystal contact. However, if most of beam values are higher than 50%, then chances are good that the interface is significant for biological assemblage. Obviously, there are many intermediate cases, which cannot be scored and interpreted unambiguously due to the reasons given above in this section. Partial probabilities in the radar are calculated using statistical distributions, derived from the properties of all interfaces calculated by *PISA* for all X-ray PDB entries (93 746 entries by the end of 2014, cf. http://www.pdb.org).

## ALGORITHMS AND SOFTWARE

### Implementation

The core computational part of *jsPISA* is built upon the standalone *PISA* software distributed by CCP4 (8), with the addition of new modules for assembly stock analysis and interaction radar. All the input/output interface and data logistics were, however, changed. The new web interface is built using the *jQuery* package (9) and uses *jqPlot, jqTree* and *D3 radar chart* libraries (10). Visualization of protein structures, interfaces and assemblies is done using the *JSMol* software (11). In contrast to *PISA* (1), *jsPISA* does not utilize parallelization on a computational cluster, which was found unnecessary given computing power delivered by modern hardware. The graphical interface was tested with modern Firefox, Safari, Chrome and Internet Explorer (MS Windows only) browsers on Mac OSX, Linux and MS Windows operating systems.

Concentrations in Equation (1) are calculated by solving the mass balance equation for chemical equilibrium (12):

(2) \begin{equation*} T_i = \sum\nolimits_j {p_{ij} K_d^j \prod\nolimits_k {c_k^{p_{kj} } } } \end{equation*}

where *T_i_* and *c_i_* are the total and free concentration of the *i*th monomeric unit, respectively; *p_ij_* is the stoichiometric coefficient of the *i*th unit in the *j*th assembly; and$K{j\atop d} = exp(-\Delta G{j\atop 0}/RT)$ is the dissociation constant of the *j*th assembly. Total concentrations *T_i_* have fixed relative values, corresponding to occurrence numbers of the corresponding monomeric units in crystal. Absolute values of *T_i_* are determined by the degree of crystal dissolution and vary from 0 to their maximum values in the crystalline state.

Solution of Equation (2) is difficult due to the extremely wide range of *K_d_* involved and requires special approaches. Our method is based on the observation that if solution {*c_i_*} for a particular set of values {*T_i_*} is known, then solution $\{ c\prime _i \}$ for $\{ (1 + \alpha )T_i \}$, where *α* is sufficiently small, may be obtained as a controllable correction $\{ \Delta c_i \} = \{ c\prime _i - c_i \}$. Such a correction may be conveniently found with an iterative bracketing procedure, where decreasing range brackets are sequentially applied to $\{ c\prime _i \}$ components. Initial values of {*c_i_*}, necessary for starting the chain of α-corrections, are calculated in the limit of sufficiently deep dissolution, where all complexes, except may be one, are fully dissociated. This procedure contains many technical details to work correctly and maintain a predefined measure of accuracy. These include, in particular, choice of α and a dynamic, rather than a preset, order of applying brackets to $\{ c\prime _i \}$ components. Full details of the method will be published elsewhere.

*jsPISA* is deployed at the CCP4 web site. It comes with detailed online documentation, available from both the home page and dynamically generated output pages. Most of the HTML elements in *jsPISA* have tooltips, which should also help understanding of the output. Therefore, only a brief description of input and output is provided in the following sections.

### Input

**Figure 2.**
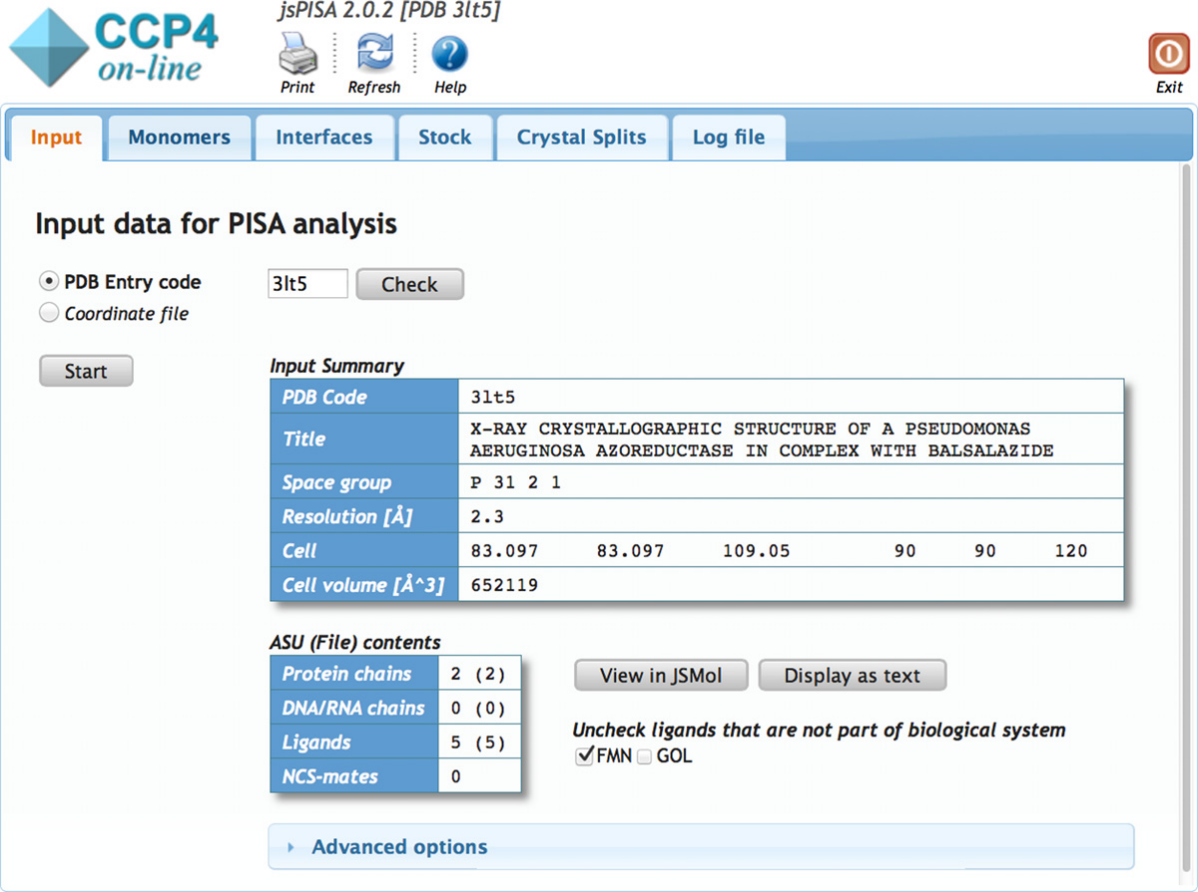
Submission form and tabbed layout in jsPISA.

Input to *jsPISA* is either a four-letter PDB entry code or a coordinate file in PDB or mmCIF (PDBx) format (cf. http://www.pdb.org), which may be also uploaded in compressed (gzipped) form. Before starting the calculations, *jsPISA* performs a preliminary analysis, aimed mostly at the identification of solvent molecules and various crystallization agents that should be removed from the system. All ligands found are listed in the submission page and those to be removed will have empty check boxes next to them (cf. Figure 2). At this point, a user may override the automatic choice and either include or exclude certain ligands in the analysis.

### Output

**Figure 3.**
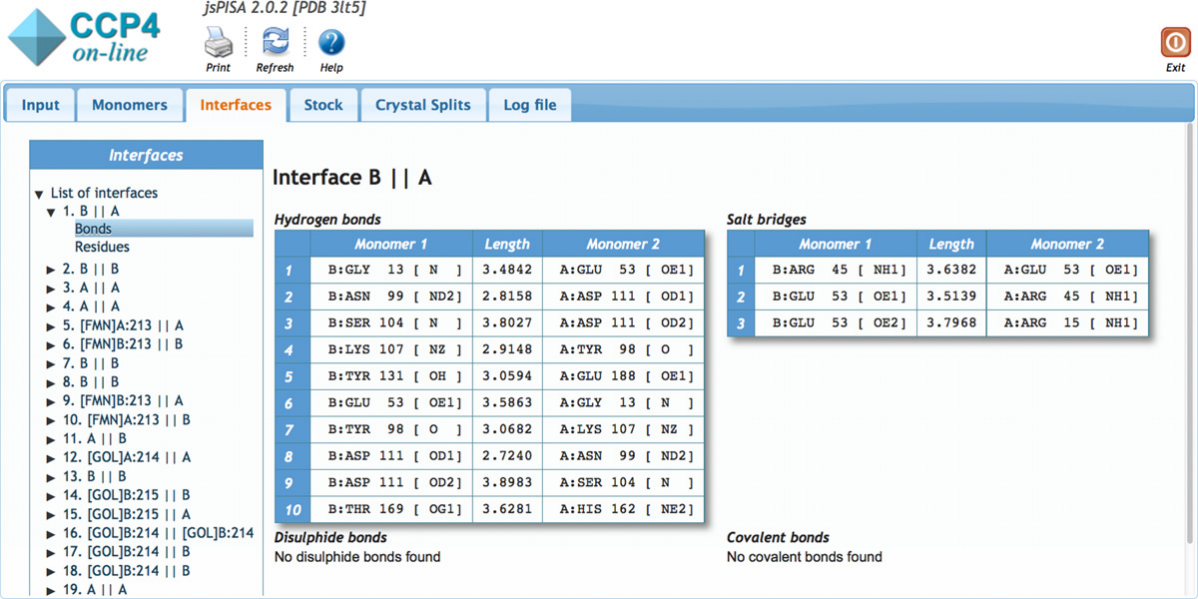
Hierarchical data layout in jsPISA.

*jsPISA* outputs the same information as *PISA* (1), augmented with the output for the new features described above. Due to the considerable volume of the output, it is arranged in a tabbed layout (see Figure 2), with separate tabs for monomer, interface, stock and crystal split data. Within tabs, the data is arranged into hierarchical structures such as *List of Interfaces* -> *Interface summary* -> *List of Chemical Bonds* -> *List of Interface Residues*, navigable with the tree widget on the left side of the output page (Figure 3). The former ‘*Assemblies*’ section of *PISA* has been renamed into ‘*Crystal Splits*’ in attempt to accentuate the meaning of the respective output. This section presents various ways to split the crystal into assemblies, with the most probable split placed on top of the list. Each split may contain more than one assembly, and it is important to realize that a whole split should be taken or rejected as a solution, and assemblies from different splits cannot be mixed up.

### Performance

Calculation times in *PISA* vary significantly, owing to graph-theoretical procedures with non-linear complexity used for the analysis of crystal lattices (1). However, most practical queries finish in <30 s. The accuracy of *PISA* results is a complex issue, which has been thoroughly investigated in (7).

## RESULTS AND DISCUSSION

*jsPISA* was tested to reproduce the results for all X-ray entries in the PDB in order to ensure that it agrees with the previous version (*PISA*) on free energy estimates and oligomeric states inferred. For this comparison, it is important to note that *PISA* does not do automatic removal of solvent molecules and crystallization agents, therefore, for getting identical results, one needs to prepend *PISA* jobs with the corresponding preparatory step.

### Assembly stock analysis

Consider a typical way of inferring the probable oligomeric state from *jsPISA* results for the example of PDB entry 3LT5. For that structure, the *Crystal Split* section in the *jsPISA* output suggests that the protein may be either homotetrameric (*A*_4_) or homodimeric (*A*_2_). Both assemblies are relatively weak, as indicated by the dissociation free energy of 3.3 kcal/mol for *A*_4_ and 10.1 kcal/mol for *A*_2_. In-depth examination of dissociation patterns and intra-assembly interfaces suggests that the tetramer dissociates into dimers, which corresponds to the chemical equilibrium

\begin{equation*} A_4 \mathop \leftrightarrow \limits^{K_d^{(1)} } 2A_2 \mathop \leftrightarrow \limits^{K_d^{(2)} } 4A \end{equation*}

where $K_d^{(1,2)} < 1$. In *PISA*, these findings would suggest that the structure is tetrameric because *A*_4_ is the highest stable structure in the dissociation chain.

**Figure 4.**
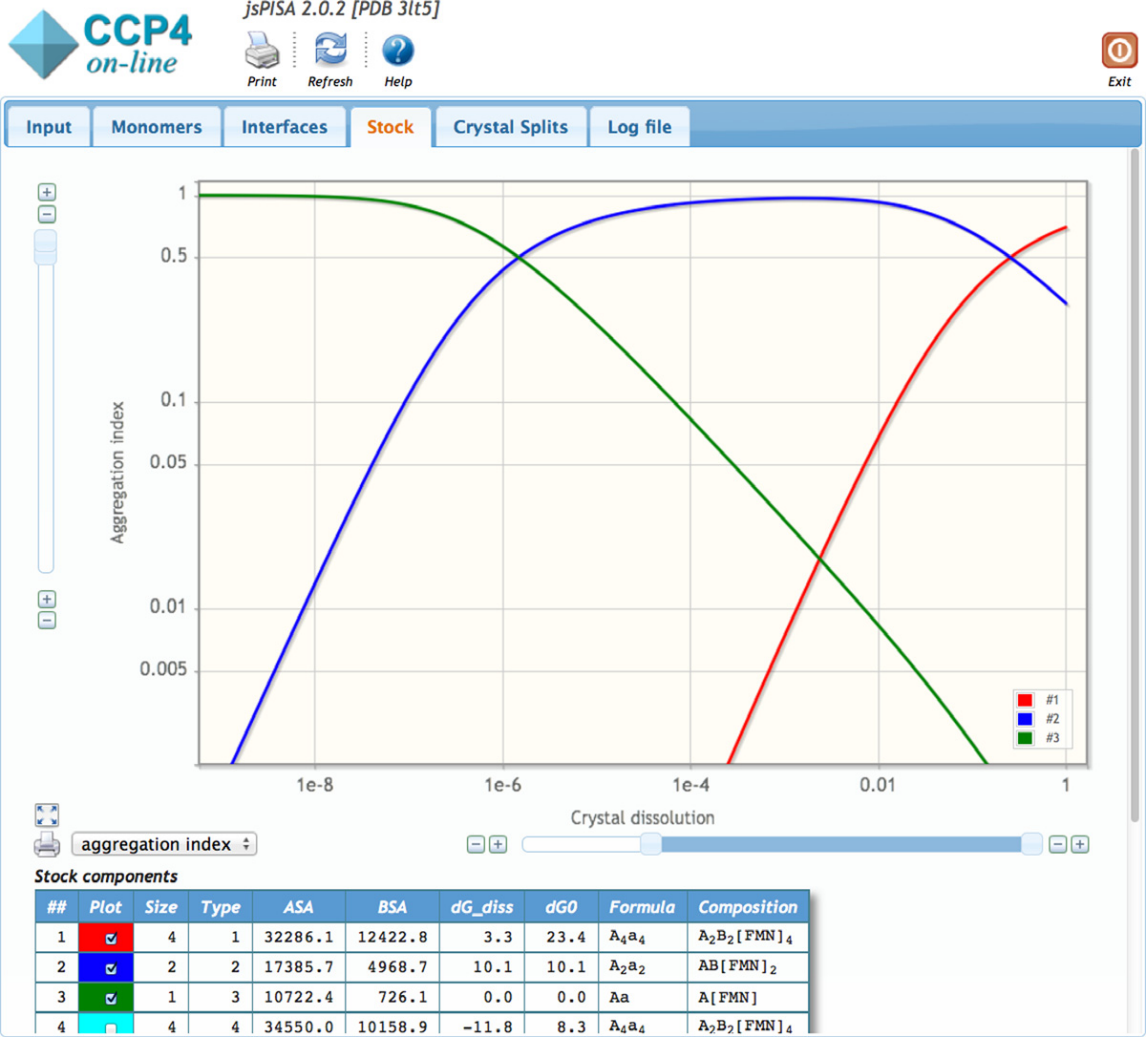
Assembly stock analysis for PDB entry 3LT5. Red line: homotetramer *A*_4_, blue line: homodimer *A*_2_, green line: monomer *A*.

However, concentration profiles in the *Stock* section of *jsPISA* output (cf. Figure 4) show that the tetramer represents the most populated aggregated state only at high protein concentrations, corresponding to >25% crystal dissolution (∼12 mM, which can be found by switching the plot from ‘aggregation index’ to ‘concentration’ mode). The co-existence of tetramers and dimers, in comparable concentrations, is observed at crystal dissolutions between 5 and 100%. At dissolutions from 0.05 to 10^–5^, up to 96% of protein exists in the dimeric form, and further dissolution prevents oligomerisation, leaving the protein in monomeric form. Therefore, it is likely that in the ‘working concentration range’ of 1 mM and lower, the protein is dimeric.

**Figure 5.**
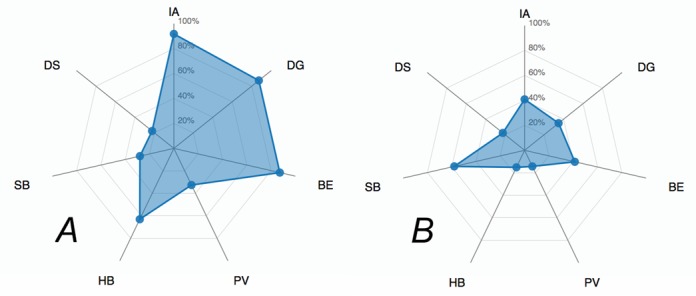
(**A**) Interaction radar for interface #1 in 3LT5, corresponding to homodimer *A*_2_. (**B**) Same for interface #2, which makes homotetramer *A*_4_ from the dimer.

The same conclusion may be also made by looking at the interaction radar for the two most significant interfaces in 3LT5. As seen from Figure 5, the radar suggests a relatively high likelihood for interface #1 (which is the homodimer) to be part of a biological assembly, in contrast to interface #2 (which makes the tetramer from the dimers). Therefore, *jsPISA* results are consistent in this particular case.

### Desktop application versus online tool

*PISA* is distributed by CCP4 as both command-prompt (*pisa*) and graphical (*QtPISA*) applications. They produce results identical to those of *jsPISA* (because they relate to standard chemical conditions, not dependent on the concentration). The command-prompt tool is convenient for using in scripted pipelines on the backyard of various bioinformatics procedures. The graphical interface of *QtPISA* is fairly similar to that of *jsPISA*, and, being a desktop application, *QtPISA* offers a somewhat higher degree of interactivity. Therefore, *QtPISA* is probably the preferable choice if the CCP4 Software Suite is already installed locally. *jsPISA* was developed and set up mainly for use by the general molecular biology and bioinformatics community, not only crystallographers, without the need to install a large and specialized crystallographic software package.

## CONCLUSION

*jsPISA* represents an advance of *PISA* software, aimed to help researchers in cases when interpretation of results is difficult due to the ambiguity of predictions or discrepancy between calculations and experimental observations. Our experience suggests that such difficulties are encountered in ∼10% of instances, often because of the uncertainty in protein concentration, at which the oligomeric state should be identified. Using concentration profiles for assembly stock in *jsPISA*, it should be easier for a researcher to check the oligomerization hypothesis by seeing whether the sought oligomer emerges within a reasonable concentration range. It should be noted, however, that there are other important parameters affecting oligomerization, such as salinity, ionic strength and pH, which are currently not taken into account explicitly. Other important factors that are currently neglected or grossly approximated in *PISA* are electrostatic interactions and entropy absorbance in low-frequency vibration modes of complexes. The corresponding modifications may be introduced in future versions of the software.
